# Strategy for the Micro-Elimination of Hepatitis C among Patients with Diabetes Mellitus—A Hospital-Based Experience

**DOI:** 10.3390/jcm10112509

**Published:** 2021-06-06

**Authors:** Pei-Yuan Su, Yang-Yuan Chen, Hsu-Heng Yen, Siou-Ping Huang, I-Ling Liu, Ya-Huei Zeng, Yu-Chun Hsu, Fu-Yuan Siao

**Affiliations:** 1 Department of Internal Medicine, Division of Gastroenterology, Changhua Christian Hospital, Changhua 50006, Taiwan; 111252@cch.org.tw (P.-Y.S.); 27716@cch.org.tw (Y.-Y.C.); 182972@cch.org.tw (S.-P.H.); 125267@cch.org.tw (I.-L.L.); 120693@cch.org.tw (Y.-H.Z.); 77149@cch.org.tw (Y.-C.H.); 2 Department of Hospitality Management, MingDao University, Changhua 523, Taiwan; 3 General Education Center, Chienkuo Technology University, Changhua 500, Taiwan; 4 Department Emergency and Critical Care Medicine, Changhua Christian Hospital, Changhua 500, Taiwan; 57385@cch.org.tw; 5 Department of Mechanical Engineering, Chung Yuan Christian University, Taoyuan 320, Taiwan; 6 Department of Kinesiology, Health and Leisure, Chienkuo Technology University, Changhua 500, Taiwan

**Keywords:** hepatitis C, screen, diabetes mellitus

## Abstract

Hepatitis C virus (HCV) infection can induce insulin resistance, and patients with diabetes mellitus (DM) have a higher prevalence of HCV infection. Patient outcomes improve after HCV eradication in DM patients. However, HCV micro-elimination targeting this population has not been approached. Little is known about using electronic alert systems for HCV screening among patients with DM in a hospital-based setting. We implemented an electronic reminder system for HCV antibody screening and RNA testing in outpatient departments among patients with DM. The screening rates and treatment rates at different departments before and after system implementation were compared. The results indicated that the total HCV screening rate increased from 49.3% (9505/19,272) to 78.2% (15,073/19,272), and the HCV-RNA testing rate increased from 73.4% to 94.2%. The anti-HCV antibody seropositive rate was 5.7%, and the HCV viremia rate was 62.7% in our patient population. The rate of positive anti-HCV antibodies and HCV viremia increased with patient age. This study demonstrates the feasibility and usefulness of an electronic alert system for HCV screening and treatment among DM patients in a hospital-based setting.

## 1. Introduction

Hepatitis C is a leading cause of cirrhosis and hepatocellular carcinoma throughout the world [1,2,3]. The newly developed direct-acting antiviral agents have revolutionized treatment for hepatitis C virus (HCV) infection compared with what was used in the interferon-based era. Direct-acting antiviral agents have displayed significantly high rates of sustained virologic response (>90%) and good treatment tolerance even in groups that are difficult to treat [2,3,4,5]. The hepatitis C care cascade from screening to treatment involves several barriers, including screening cost, patient awareness, and the lack of adequate manpower. Therefore, a micro-elimination approach targeting specific high-risk populations is less complex and less costly than a universal screening and treatment approach [6,7]. Several specific high-risk populations with bloodstream infections have been identified as the first step toward HCV elimination, including injection drug users [8], HIV-infected patients [2,9], and patients on dialysis [3].

Hepatitis C can induce liver-related complications and is associated with many extrahepatic manifestations such as insulin resistance, diabetes mellitus (DM), renal insufficiency, cryoglobulinemia, and lymphoma [10]. Previous studies demonstrated a higher prevalence of HCV infection among patients with diabetes [11,12,13]. In a community-based 10-year follow-up study, Lin et al. [14] found a hazard ratio of 1.53 of developing DM among an anti-HCV seropositive population during 180,244 person-years of follow-up. Chen et al. [13] reported anti-HCV seropositive rates of 6.8% among DM patients and 2.6% among a control group in Taiwan. A diabetes management quality program from the National Health Government in Taiwan that requires patients to undergo biannual check-ups for various metabolic measurements to improve the care quality has been established for years [15]. Therefore, implementing an HCV screening and treatment strategy targeting this population during their outpatient clinic visits is feasible and may facilitate achieving the goal of an 80% treatment coverage rate with direct-acting antiviral agents by 2025 in Taiwan [16,17]. In this study, we aimed to report our outcomes of utilizing an electronic alerting system for HCV screening among patients with a diagnosis of DM in a tertiary referral center.

## 2. Materials and Methods

### 2.1. Patient Identification

Patients with a diagnosis of DM who were receiving management in the outpatient department were identified as candidates for HCV screening and treatment in our hospital from August 2019 to December 2020 in response to the government policy for HCV elimination [1,16]. Eligible patients fulfilled the following criteria: (1) a diagnosis of DM based on the ICD-10-CM codes (E08-E13) and (2) regular follow-up for the past six months in our hospital.

### 2.2. Electronic Alerting System

**Figure 1:**
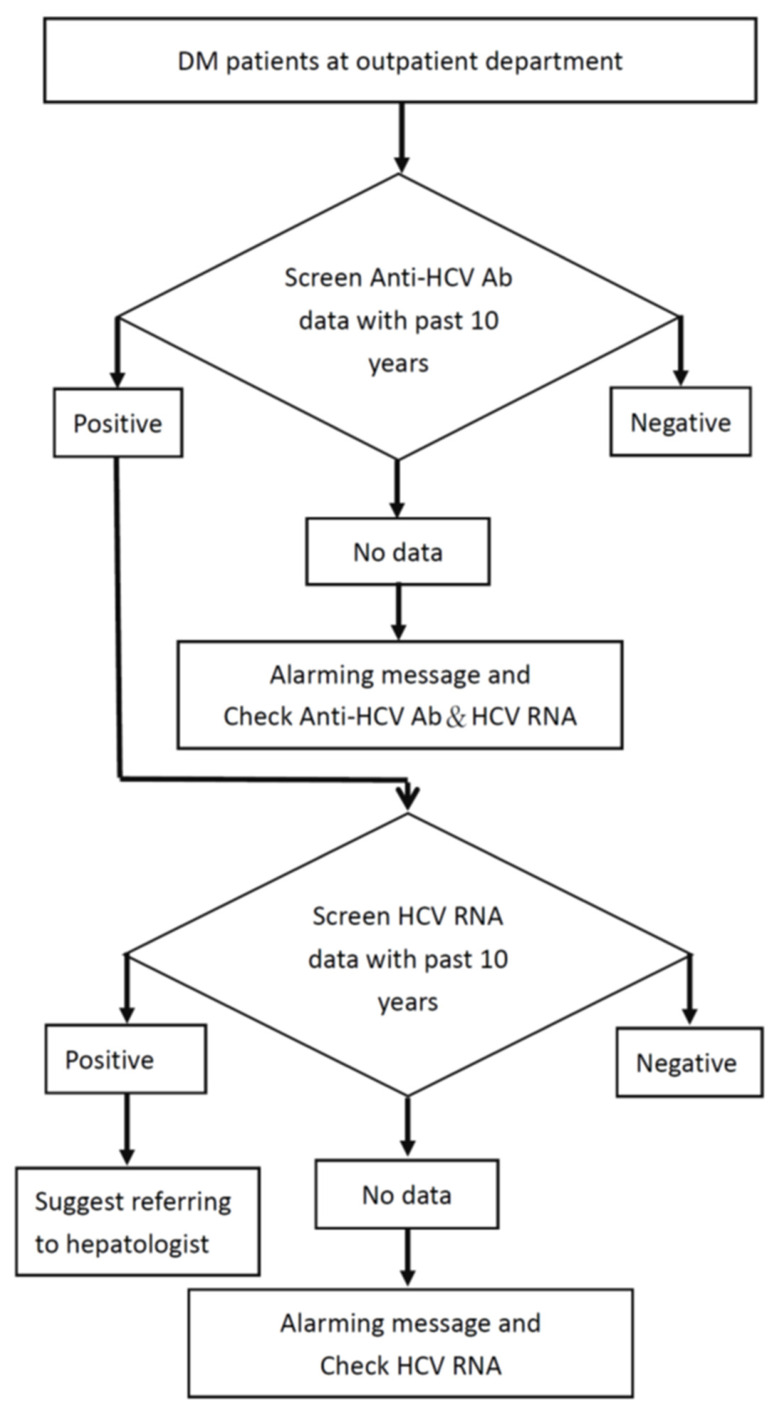
Hepatitis C virus screening flowchart targeting the diabetes mellitus population.

Figure 1 illustrates the electronic alerting system that was developed and has been used since August 2019 in the hospital. Once the DM patient visits the clinic with a planned check-up for metabolic measurements, the system automatically searches the laboratory database of the hospital for HCV-related testing in the past decade in the background. When the clinician opens the patient data, an automatic pop-up screen appears according to the result of computing (Figure 1), and the physician needs one click to generate the order. The screening rate is defined as the number of patients with test results for anti-HCV antibodies (HCV-Abs) of all eligible patients.

In the first step, the system checks the HCV-Ab status and recommends that the physician order reflex anti-HCV testing (including HCV-Ab and HCV-RNA testing) in case there are no previous HCV-Ab results after obtaining patient permission [1,18]. Next, if the patient was positive for HCV-Abs but has no available HCV-RNA testing data, alarm messages would recommend that the physician order HCV-RNA testing after obtaining patient permission. Third, if the patient had positive HCV-RNA test results, a referral sheet to a hepatologist is automatically printed out that facilitates the patient’s transfer for subsequent HCV therapy.

Phase 1, the introduction phase of utilizing the electronic alert system, lasted from August 2019 to June 2020 for patients who visited diabetes clinics and the nephrology department. In phase 2, the implantation phase, the electronic alert system was extended to all outpatient departments in the hospital; this phase lasted from July 2020 to December 2020.

### 2.3. HCV Testing

HCV-Abs were tested for using the ARCHITECT anti-HCV assay (Abbott Laboratories, Abbott Park, IL, USA). HCV RNA was quantitatively measured using ART HCV assays (RealTime HCV test, Abbott Molecular, Abbott Park, IL, USA) [19].

### 2.4. Ethical Considerations

The Ethics Committee of Changhua Christian Hospital approved the study protocol (CCH IRB No: 200403), and informed consent was waived because the research was designed to study and evaluate a public program subject to the approval of our local government.

### 2.5. Statistical Analysis

Cochran’s Q test was utilized to compare the screening rates at different time periods with post hoc test using Dunn’s test, which applies Bonferroni correction. All statistical data were analyzed using SPSS software version 18.0 (SPSS Inc., Chicago, IL, USA), with *p* < 0.05 indicating statistical significance.

## 3. Results

### 3.1. HCV Screening Rate

A total of 19,272 patients with a diagnosis of DM who fulfilled the inclusion criteria were identified in August 2019. The patients had a mean age of 65.6 years and were predominately male (52.77%) (Table 1). There were 10,235 (53.1%), 907 (4.7%), 1072 (5.6%), and 7058 (36.6%) patients with DM in diabetes, nephrology, gastroenterology, and other departments, respectively.

The baseline HCV screening rate before the introduction of the electronic reminding system was 49.3%, which increased to 73.9% after phase one and 78.2% after phase two implementation. The baseline HCV screening rates were 48.4%, 67%, 64.2%, and 46.1% in the diabetes, nephrology, gastroenterology, and other departments, respectively. After the phase one screening period (10 months), the HCV screening rate increased to 90.5%, 86.5%, 67.4%, and 49.3% in the diabetes, nephrology, gastroenterology, and other departments, respectively. The screening rate further increased to 91%, 88.3%, 71.8%, and 59.4% in the diabetes, nephrology, gastroenterology, and other departments after the phase two screening period, respectively. The HCV-RNA testing rate was 73.4% at baseline, which increased to 87.8% after phase one and 94.2% after phase two implementation.

### 3.2. HCV Testing Results

**Figure 2:**
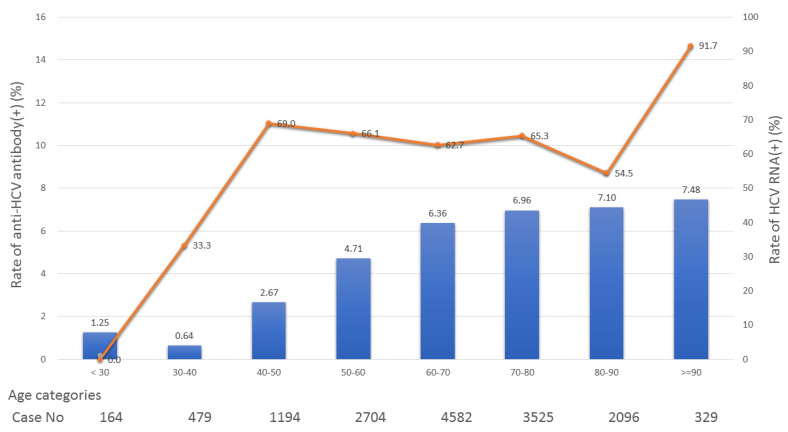
The associations between seropositive hepatitis C virus viremia, antibody rate, and age.

Figure 2 shows the positive rates of HCV-Abs and HCV RNA according to patient age group. The positive rates of HCV-Abs and HCV-RNA testing increased among elderly patients. The highest positive rate was 7.48% in patients older than 90 years old, whereas the lowest positive rate was less than 2% in patients younger than 40 years old. The HCV RNA positive rate was higher in those aged 40 years and older compared with those younger than 40 years old.

### 3.3. Cascade of HCV Screening and Treatment

**Figure 3:**
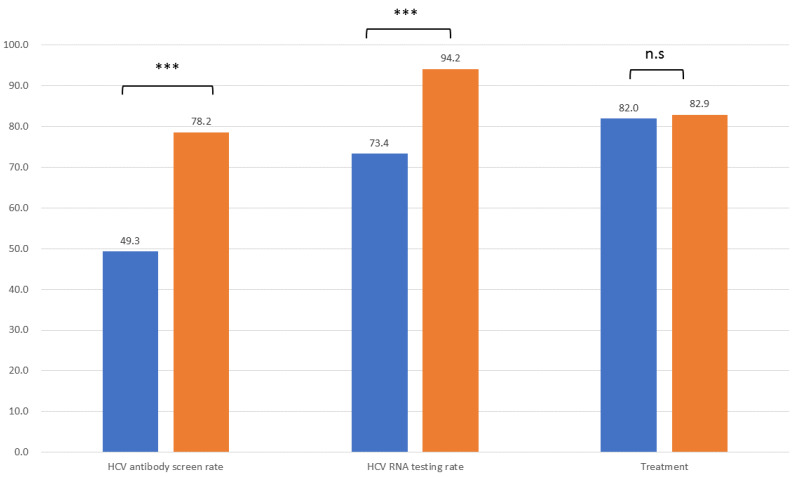
The HCV-Ab screening rate, HCV RNA testing rate, and HCV treatment rate before (blue bar) and after (orange bar) the micro-elimination program. *** *p* < 0.0001, n.s.: non-significant.

The final HCV screening rate for HCV-Abs was 78.2% in patients with DM in our hospital during the study period, and the positive rate for HCV-Abs was 5.7%. HCV RNA was confirmed in 94.2% of patients who had positive HCV-Abs, and the positive rate of HCV RNA was 62.7%. Finally, 82.9% of patients with positive HCV RNA received direct-acting antiviral therapy in the hospital (Figure 3). A significantly increased HCV Ab screening rate and HCV RNA testing rate but not treatment rate was observed after this micro-elimination program (Figure 3).

## 4. Discussion

As far as we know, this is the first study evaluating an HCV micro-elimination approach targeting patients with a diagnosis of DM that utilizes an electronic alert system in a hospital-based system. The overall HCV-Ab screening rate in this patient population was 78.2%, and 94.2% of the HCV-Ab positive patients received subsequent HCV-RNA testing. A total of 82.9% of patients with positive HCV-RNA testing received direct-acting antiviral therapy in the hospital.

Universal screening is an ideal but not cost-effective strategy toward HCV elimination in most clinical settings [20,21]. In the United States, only 20% of the 3.5 million HCV-infected patients were screened, 27% were tested for HCV-RNA, and 9% were treated [22]. The effective identification of high-risk patients for HCV infection is the first step toward HCV elimination, and such a micro-elimination approach is beneficial for clinicians and public health officials due to limited financial support and the shortage of screening manpower [23]. Several high-risk subpopulations, such as injection drug users [8], prisoners [24], HIV-infected patients [2,9], dialysis patients [3], and baby boomers [18,25] have been reported as the first target for HCV elimination. Despite the DM population being identified as having a higher risk for hepatitis C infection than the general population [13,26,27,28], these patients have not been elected as a target for HCV micro-elimination in previous literature. Since these patients require regular blood testing for their liver function and metabolic profile during clinical follow-up [29], screening these patients without requiring additional blood sampling during their scheduled clinic visit is a feasible approach for HCV elimination, as demonstrated in the current study. In addition, a baseline high screening rate in our population also accounts for the successful implementation of the system in the present study.

Despite the defined high-risk population for HCV screening, implementing such a screening process is difficult even in hospitals given healthcare professionals’ lack of knowledge and/or lack of interest in patient referral, particularly among non-hepatology departments [30]. The utilization of an electronic alert system has been reported to improve the screening and referral rate for the eligible population in the hospital [1,30]. Konerman et al. [31] developed an alert system for HCV screening that dramatically improved the screening rate from 7.6% to 72% over a one-year period. Morales-Arraez D et al. [32] utilized electronic alert messages to increase the HCV-RNA testing rate from 62.4% to 77.7% and shortened the time lag between RNA testing and positive HCV antibody testing from 19.1 to 6.6 months. Our algorithm not only automatically identifies the eligible population during the clinic visit but also recommends lab examination forms automatically for the physician. Additionally, we utilized a reflex testing strategy in our algorithm [1] for those who had never been tested for HCV-Abs that explained the high HCV-RNA testing rate in our study. Although the electronic alert system can help clinicians order HCV-Ab or HCV-RNA tests, the screening rate varied between different departments in the present study. The HCV screening rate is more likely to increase for patients visiting diabetes medicine and nephrology clinics. The increase was modest in the gastroenterology department and other departments. Possible explanations may be related to the need for regular follow-up for patients visiting diabetes medicine or nephrology clinics and because the physicians providing such care are educated regarding the beneficial effect of anti-HCV therapy for these patients. Patients with a DM diagnosis who visit other clinics may have less regular follow-up or more co-morbidities, such as cirrhosis, malignancy, or stroke, and their clinicians may not order further laboratory tests for these patients.

The overall positive rate of HCV-Abs among our DM patients was 5.7%, which is higher than the previously reported 2.8% prevalence among healthy volunteers in Changhua [33]. The higher age of our patient population may explain the high HCV prevalence with different age strata than the general population. Furthermore, higher HCV rate in DM patients reflects the close association of chronic hepatitis C infection and insulin resistance [10,34,35]. Additionally, DM is known to increase the risk of HCV-related complications such as hepatocellular carcinoma [10,36]. Identifying DM patients with unrecognized HCV infection may not only help improve their glycemic control [37] but also further lowers their risk of developing liver-related complications [4,38,39] after successful HCV eradication. Through the screening and referral program in the present study, we could achieve a higher HCV screening and treatment rate than those in previous reports [1,40,41]. Such an approach may pave the way for improved HCV care and is key to facilitating the HCV care cascade, particularly for the target patient population from non-hepatology departments in the present study. The treatment uptake of our patients was significantly increased, indicating the success of the model in facilitating in-hospital HCV micro-elimination for DM patients.

There are some limitations in our study. First, our electronic alerting system that helps clinicians order HCV testing is not mandatory. We did not record the reasons why clinicians or patients did not opt for HCV screening tests for further analysis. The interest of the physician regarding hepatitis C care, patient co-morbidities, or patient awareness of the disease may affect the HCV screening rate. Second, some patients may have received previous anti-HCV therapy in other hospitals, accounting for the lower HCV viremia rate in the present study [1]. Third, we did not collect patient data such as lipid profiles for further analysis of their association with HCV infection.

## 5. Conclusions

In the present study, we found that using an electronic system for HCV screening for patients with DM is a feasible and effective approach toward HCV elimination. It can be a useful way to achieve HCV micro-elimination in this population, which has a higher seropositive rate of HCV-Abs.

## Figures and Tables

**Table 1:** Comparison of screening rates at different time points according to the outpatient department.

	Patient Number	Baseline	Phase 1	Phase 2	Cochran’s Q Test		Post Hoc Test		
					χ^2^	*p*-Value	*p*-Value ^a^	*p*-Value ^b^	*p*-Value ^c^
Screened Patient Number	19,272	9505(49.3%)	14251(73.9%)	15073(78.2%)	9734.703	***	***	***	***
Diabetes medicine	10,235	4955(48.4%)	9261(90.5%)	9310(91.0%)	8613.103	***	***	***	1
Nephrology	907	608(67.0%)	785(86.5%)	801(88.3%)	356.653	***	***	***	0.475
Gastroenterology	1072	688(64.2%)	723(67.4%)	770(71.8%)	123.878	***	***	***	***
Others	7058	3254(46.1%)	3482(49.3%)	4192(59.4%)	1530.84	***	***	***	***

Note: a. *p*-value for Baseline vs. Phase 1; b. *p*-value for Baseline vs. Phase 2; c. *p*-value for Phase 1 vs. Phase 2; *** *p* value <0.001.
